# Supplementary material for: Caregiver perceptions and experiences of paediatric emergency department attendance during the COVID-19 pandemic: A mixed-methods study
Source: PLoS One. 2022 Nov 16;17(11):e0276055. doi: 10.1371/journal.pone.0276055 (PMC9668109; doi:10.1371/journal.pone.0276055)
Supplement: S1 File — (DOCX) [file pone.0276055.s001.docx]

Partial Date of birth (MM:YR) of child/ young person: _____/_____ Sex of child/ young person:  __________________

Partial Date of birth (MM:YR) of parent/guardian/ legal representative: _____/____

Sex of parent/guardian/ legal representative:  _____________ First part of Postcode (eg SW13):  ______________

Ethnicity of parent/guardian/ legal representative: ________________

1. On a scale of 0 to 10 please **circle** how worried were you about coming to hospital today?

(*least worried*) **0 1 2 3 4 5 6 7 8 9 10** (*most worried*)

1. If you were worried, was it because… (Tick all that apply)

🞎 of anxiety about contracting the Coronavirus (COVID-19)?

🞎 you were not sure if the Children’s Emergency Department was open as usual?

🞎 you were worried about over–using the service?

🞎 you were worried about breaking lockdown rules?

🞎 of messages on social media?

🞎 Other? Please write below.

_______________________________________________________________________________________

1. If there wasn’t a COVID-19 pandemic would you have: (please tick)

🞎 Attended Paediatric emergency Department (PED) earlier?    🞎 Seen your GP?

🞎 Remained at home?       🞎 Not attended PED at all?

🞎 Other? Please write below.

_______________________________________________________________________________________

1. Why have you brought your child today? (Tick all that apply)

🞎 Cough 🞎 Fever

🞎 Cold or Flu like illness 🞎 Mental health issue

🞎 Vomiting and / or diarrhoea 🞎 Injury

🞎 Other? Please write below.

_______________________________________________________________________________________

1. For how long has your child been unwell? (please tick)

🞎 Less than 24 hours 🞎1-2 days 🞎 3-5 days 🞎over 5 days

1. Did you discuss your concerns with any other medical professional before you came to hospital? (please tick)                     🞎  Yes 🞎 No

**PTO**

1. If yes, you did discuss your concerns with any other medical professional who? (please tick all that apply)

🞎 GP                        🞎 111

🞎 Midwife                     🞎 Pharmacy

🞎 On-line advice 🞎 Nurse-led service

🞎 Other? ____________________________________________________________________

1. If yes, did they recommend coming to the PED? (please tick)

🞎Yes 🞎 No

1. How did you come to Hospital today?

🞎 Own Vehicle 🞎 Public Transport 🞎Ambulance

🞎 Other _____________________________________________________________________

**Thank you for taking part**

Please give this completed questionnaire back to a member of the research team

**For medical staff to complete.**

Date:

Triage category on arrival:

Final diagnosis:

Treatment needed? Yes / No

If yes, what?

Any underlying health problem?

If yes, what?

Outcome?: Home / Admitted to PSSU / Admitted to the inpatient ward / Retrieved?

Length of attendance in A+E?
