# Supplementary material for: Caregiver perceptions and experiences of paediatric emergency department attendance during the COVID-19 pandemic: A mixed-methods study
Source: PLoS One. 2022 Nov 16;17(11):e0276055. doi: 10.1371/journal.pone.0276055 (PMC9668109; doi:10.1371/journal.pone.0276055)
Supplement: S2 File — (DOCX) [file pone.0276055.s002.docx]

# Face to face Telephone Video Call

“Thank you for agreeing to take part in this interview. If you agree, we are going to use a device to record you this is just to ensure we capture everything they say, as writing notes may miss something, and makes it easier to fully listen to them, but will be deleted after being written. You can decide to stop at any time or choose which questions to answer”

1. How are you feeling about this COVID-19 pandemic?

(probe specific to question, if not covered) What were your initial thoughts?

(probe specific to question, if not covered) Have they changed now?

1. What are or were your thoughts about attending hospital during this period (COVID -19)? lockdown

(Probe specific to question, if not covered) In the beginning?

(probe specific to question, if not covered) Are your thoughts different now and why?

What fears or concerns did you have about attending hospital? Probe where these fears/concerns came from/where heard such… Probe how overcame such fears (strategy/plan? What reassured fears?)

What concerns have you heard from other parents/ guardians/ legal representatives /members of the public, about bringing children to hospital during COVID-19?

What drove you to attend today? What triggered/influenced that decision?

Have there been other times during COVID-19 that you considered coming to hospital? Did you come? Why/why not?

1. What information did you receive about attending PED or Hospitals during the COVID-19 pandemic?

1. What did you think about this information?
2. Where did you receive this information from?

(probe specific to question, if not covered) Is that information different now?

(probe specific to question, if not covered) How is the information different now?

6. How do you think information regarding hospital or ED attendances should be made available to the public?

(Probe who should be targeted and why e.g. if not covered ….

A: Who should the information go to?

B: Why do you think that?

C: Do you think information dissemination has improved recently and why?

7. How should information about the seriousness of illness and when to attend hospital be presented to the public?

Why do you think this?

Have you received any information regarding this previously or in present times?

1. What do you think the role of parent/ guardian/ legal representative should be in the assessing the seriousness of illness and need to attend ED?

9. Where do you think it would be best to share information about attending hospital during the pandemic? E.g. through GP surgeries, the media, hospital Doctors and 111 services should be in the dissemination of this information to the public? (Probe: How each group mentioned above should be encouraged to take an active role in the dissemination of this information?) (see page 3 for groups)

Hospital doctors?

GP Surgeries?

111 services

The media?

The government?

10 . What could be done to encourage community members to participate in sharing this information?

11. Do you have suggestions or ideas for addressing possible delays in presentation to ED due to community/public concerns mentioned above in the current Covid-19 Pandemic?

B. Perceptions about time to be reviewed

12. From the time that your child arrives in PED/ PAU how long would you be willing to wait for medical review? (<30 minutes, 30 minutes to 1 hour, 1 to 2 hours).

Explain why?

Possible probe how has the COVID-19 pandemic shaped this? How has this changed during COVID-19? Has this affected how long you are comfortable or willing to wait? (during this lockdown do you think you should wait longer or shorter and why?)

C. Decision making

13. What influenced your decision to attend the hospital during these times (of COVID-19)?

Who did you discuss this decision with?

What information did you seek in making your decision?

(Only ask this question if not mentioned in previous answer)

14. How would you like the hospital to provide you with information about patients with COVID1-19 in the department.

15. Would you encourage other parents/ guardians/ legal representatives to bring their children to ED if they were ill in this COVID -19 pandemic?

Yes □ No □

If no, what would be your main concerns and worries?

16. Do you have any other thoughts you wish to share on this topic?

Do you have any (other) recommendations for how things can be improved

Do you have any questions for me?

*Thank you for taking part*

**Prompts that may be used by the researcher include:**

Can you tell me more…? / Do you have an example of that…? Can you elaborate a bit …?

Why do you say that/ what makes you say that? Mm, and other non-verbal cues such as use of eye contact, head nodding
