# Supplementary material for: Caregiver perceptions and experiences of paediatric emergency department attendance during the COVID-19 pandemic: A mixed-methods study
Source: PLoS One. 2022 Nov 16;17(11):e0276055. doi: 10.1371/journal.pone.0276055 (PMC9668109; doi:10.1371/journal.pone.0276055)
Supplement: S3 File — Table A Attendance figures compared to study data. Table B Responses to questionnaire compared between the two groups. Table C Quantitizing of the qualitative responses. Table D Quantitizing of qualitative responses around sources of information. (DOCX) [file pone.0276055.s003.docx]

# Supplementary data: Qualitative and Quantitative Results

## Quantitative

**Table A:** Attendance figures compared to study data

Figures in 2019 and 2020 relate to a 4-month time period between September and December. Our study findings represent a seven-week period between November – December 2020 of data collected between 09:00 – 17:00

|  | **Category** | **Figures 2019**  **N= 19165** | **Percentage of 2019** | **Figures 2020**  **N=10,357** | **Percentage of 2020** | **Our study figures**  **(N=100)** | **Percentage of our study findings** |
| --- | --- | --- | --- | --- | --- | --- | --- |
| **Common reasons for presentation** | Injury | 2902 | 24% | 2122 | 32% | 29 | 29% |
|  | Respiratory | 3144 | 26% | 1935 | 16% | 11 | 11% |
|  | Gastrointestinal | 1209 | 10% | 530 | 8% | 11 | 11% |
|  | Mental Health/ Self Harm | 121 | 1% | 133 | 2% | 3 | 3% |
| **Arrival mode/ Transport** | Own / Walk in - lift with family member, school car | 13978 | 73% | 7746 | 74% | 64 | 64% |
|  | Public transport / Taxi | 2691 | 14% | 1037 | 10% | 19 | 19% |
|  | Ambulance | 2488 | 12.9% | 1564 | 15% | 13 | 13% |
|  | Police Transport | 8 | <1% | 10 | <1% | 0 | 0% |
|  | Not given | - | - | - | - | 6 | 6% |

**Table B:** Responses to questions asked in the questionnaire compared between two groups to show similar characteristics

|  | **Category** | **Questionnaire Response Only**  **(N=80)** | **Questionnaire & Interview**  **(N=18)** | **Total** |
| --- | --- | --- | --- | --- |
| **Worry level** | 0 | 13 | 5 | 18 |
|  | 1 | 7 | 1 | 8 |
|  | 2 | 13 | 1 | 14 |
|  | 3 | 7 | 2 | 9 |
|  | 4 | 2 | - | 2 |
|  | 5 | 5 | 1 | 6 |
|  | 6 | 6 | 3 | 9 |
|  | 7 | 9 | 4 | 13 |
|  | 8 | 8 | 1 | 9 |
|  | 9 | 6 | - | 6 |
|  | 10 | 4 | - | 4 |
| **Median Worry Level** |  | 3.5 | 4 | 3.5 |
| **Reason for worry level**  **(N=123)^[[1]](#footnote-2)^** | Not worried | 17 | 5 | 22 |
|  | COVID anxiety | 31 | 7 | 38 |
|  | Overuse worry | 20 | 7 | 27 |
|  | Unsure of opening | 10 |  | 10 |
|  | Other - not expanded | 9 | 1 | 10 |
|  | Other - worry about child | 8 | 1 | 9 |
|  | Other - thought it might be busy / long waiting time | 2 | 1 | 3 |
|  | Worry of breaking rules | 3 |  | 3 |
|  | Wasting staff time | 1 |  | 1 |
| **Illness duration** | <24 hours | 38 | 8 | 46 |
|  | 1-2 days | 12 | 4 | 26 |
|  | 3-5 days | 14 | 3 | 17 |
|  | 5+ days | 16 | 3 | 19 |
| **Course of action if pandemic not occurring** | Attended earlier | 25 | 6 | 31 |
|  | Seen GP | 13 | 3 | 16 |
|  | Same time to attend | 35 | 7 | 42 |
|  | Not attended | 1 | 0 | 1 |
|  | Other | 6 | 2 | 8 |
| **Pre-attendance discussions with a health professional^[[2]](#footnote-3)^** | GP | 21 | 7 | 28 |
|  | GP receptionist | 1 | 2 | 3 |
|  | 111 | 14 | 5 | 19 |
|  | Ambulance/ 999 | 4 | 1 | 5 |
|  | Hospital/ point of contact to service | 4 | - | 4 |
|  | Midwife | 3 | - | 3 |
|  | School | 2 | - | 2 |
|  | Medical friend |  | 1 | 1 |
|  | Health Visitor | 1 | - | 1 |
|  | Pharmacy |  | 1 | 1 |
|  | Online | 1 | - | 1 |
|  | Not answered | 2 | - | 2 |

## Mixed Methods

## Table C: Mixed Methods quantitizing of qualitative data.

The first two rows are quantitative data from the survey from the participants. The remaining rows are quantitized qualitative responses.

|  | | **Level of Worry (LOW)** | | | | |
| --- | --- | --- | --- | --- | --- | --- |
|  |  | **Low 0-3**  **N=9** | **Medium 4 – 6 N=4** | **High**  **7 –10**  **N=5** | **Not stated**  **N = 2** | **Total** |
| **Illness Duration for current presentation**  **(Quantitative)** | <24 hours | 6 | 1 | 1 | - | 8 |
|  | 1 -2 days | 2 | 1 | 1 | - | 4 |
|  | 3 -5 days | 1 | 1 | 1 | - | 3 |
|  | More than 5 days |  | 1 | 2 | - | 3 |
| **Behaviour if no pandemic (Quantitative)** | Other | 2 |  |  | - | 2 |
|  | No change – same behaviour | 5 |  | 2 | - | 7 |
|  | Seen GP | 2 | 1 |  | - | 3 |
|  | Attended Earlier |  | 3 | 3 | - | 6 |
| **Attended earlier in the pandemic** | Yes attended hospital | 3 |  |  |  | 3 |
|  | Yes sought alternative care |  |  | 1 |  | 1 |
|  | No need to attend | 3 | 3 | 4 | 2 | 12 |
|  | Not stated | 3 | 1 |  |  | 4 |
| **Trigger** | Parent sought advice from 111/GP/Other | 6 | 2 | 4 | 1 | 13 |
|  | Perceived severity led directly to attendance | 3 |  |  |  | 3 |
|  | Exhausted primary care and feel need secondary care |  | 2 |  |  | 2 |
|  | School suggestion |  |  | *1* | 1 | 2 |
| **Role of Caregiver** | Parent Instinct/ Gut instinct | 6 | 1 | 1 | 1 | 9 |
|  | Role of CG attributable to experience from other children | 2 |  |  |  | 2 |
|  | Role of CG experience from training | 1 | 1 | 1 |  | 3 |
|  | Follow advice |  | 3 | 3 | 1 | 7 |
| **Reason for Worry^1^** | Anxiety around COVID-19 | 3 | 4 | 4 | 2 | 13 |
|  | Overburden the NHS | 1 | 2 | 1 |  | 4 |
|  | Transmission risk of covid on public transport | 2 | 1 |  | 1 | 4 |
|  | Not worried | 4 |  | 1 | 1 | 6 |

Some participants gave >1 response

**Table D: Quantitizing qualitative information around sources of information**

|  | | **Level of Worry (LOW)** | | | | |
| --- | --- | --- | --- | --- | --- | --- |
|  |  | **Low 0-3**  **N=9** | **Medium 4 – 6 N=4** | **High 7 –10**  **N=5** | **Not stated**  **N = 2** | **Total** |
| **Recalled if received Information** | Yes | 3 | 1 | 1 | 1 | 6 |
|  | No | 5 | 1 | 2 | 1 | 9 |
|  | Not stated | 1 | 2 | 2 |  | 5 |
| **Sources of information**  **mentioned** | 111 | 6 | 3 | 3 | 1 | 13 |
|  | GP services: GP/ GP text/ GP reception | 7 | 1 |  | 1 | 9 |
|  | Pharmacy |  | 1 | 1 |  | 2 |
|  | Doctors – hospital based | 1 | 1 |  |  | 2 |
|  | Government briefings | 2 | 2 | 1 | 1 | 6 |
|  | Government Website | 2 |  |  |  | 2 |
|  | Whats App/ Social Media | 4 | 2 |  | 1 | 7 |
|  | Mainstream media television (TV) | 4 | 1 | 2 |  | 7 |
|  | Radio | 2 |  | 1 |  | 3 |
|  | School | 1 |  | 1 | 1 | 3 |
|  | Friend | 2 | 2 | 1 |  | 5 |
|  | Family | 2 | 1 |  |  | 3 |
|  | Internet | 3 | 3 | 2 | 1 | 9 |
|  | No one else | 1 | 1 |  |  | 2 |
| **Imagined ways to share information** | 111 | 3 | 1 | 2 | 1 | 7 |
|  | GP services: GP/ GP text/ GP reception | 3 | 1 | 4 | 1 | 9 |
|  | Pharmacy | 1 |  |  |  | 1 |
|  | Drs – non GP |  | 1 | 2 |  | 3 |
|  | Midwife | 2 | 1 | 2 |  | 5 |
|  | Health visitor | 2 |  | 1 | 1 | 4 |
|  | District Nurse | 1 |  |  |  | 1 |
|  | Whats App/ Social Media | 5 | 2 | 1 | 1 | 9 |
|  | Mainstream media TV | 4 | 2 | 2 |  | 8 |
|  | Radio | 1 |  | 2 |  | 3 |
|  | Posters/ billboard/ public space/ community spaces | 5 | 2 | 1 | 1 | 9 |
|  | School | 2 | 2 | 2 | 1 | 7 |
|  | Word of Mouth | 1 | 1 | 1 | 1 | 4 |
|  | Government Website/ Prime minister | 1 |  |  |  | 1 |
|  | Internet | 3 | 3 | 2 | 1 | 9 |
|  | Not a leaflet | 3 |  |  |  | 3 |

1. Some participants gave >1 response [↑](#footnote-ref-2)
2. 70 contacts by 66 participants [↑](#footnote-ref-3)
