# Supplementary material for: Caregiver perceptions and experiences of paediatric emergency department attendance during the COVID-19 pandemic: A mixed-methods study
Source: PLoS One. 2022 Nov 16;17(11):e0276055. doi: 10.1371/journal.pone.0276055 (PMC9668109; doi:10.1371/journal.pone.0276055)
Supplement: S4 File — (DOCX) [file pone.0276055.s004.docx]

**Good Reporting of a Mixed Methods Study (GRAMMS) checklist**

File adapted from Gugsa *et al* [1] and checklist items taken from O’cathain *et al* [2].

| (1) | **Describe the justification for using a mixed methods approach to the research question**  A mixed-methods approach enabled us to quantify changes to attendances to paediatric emergency care as well as to understand why and how caregivers may be changing their health-seeking behaviour and decision-making because of the pandemic. The rationale for the study design is further outlined in the Material and Methods section. |
| --- | --- |
| (2) | **Describe the design in terms of the purpose, priority and sequence of methods**  Both surveys and semi-structured interviews were conducted concurrently and at a single time point although the participation in each study was optional. This was important due to the dynamic nature of the pandemic. Surveys were administered before interviews due to time constraints as the study was conducted alongside and between clinical care being delivered. Therefore, there are differing number of participants across each study method. Further detail is given in the Material and Methods section. |
| (3) | **Describe each method in terms of sampling, data collection and analysis** |
|  | This is detailed in the Material and Methods section under relevant subheadings. Specifically, pages 8-10 outline data collection and pages 10 -11 data analysis. |
| (4) | **Describe where integration has occurred, how it has occurred and who has participated in it**  Integration occurred during the interpretation phase of the study and was completed by the joint first authors (VP, GA) and co-authors (SH, AWS, ME, MNN). GA also integrated the data during the analysis (see the Results section under the subheading (Mixed Methods Results)). This has been cross-checked by VP – see pages 33-38. |
| (5) | **Describe any limitation of one method associated with the present of the other method**  Presenting the quantitative data alone would prevent us from understanding the enabling factors and perceived barriers to seeking healthcare, and specifically the underlying motivations behind this. Qualitative research provides the context behind decisions by caregivers to present, or delay presenting, to emergency paediatric care. Further limitations are presented in the Discussion section. |
| (6) | **Describe any insights gained from mixing or integrating methods** |

By integrating findings from each method, we were able to determine that the most significant point of delaying access to care was made at the decision to seek care. This decision by caregiver was influenced by intrinsic factors such as “parental intuition” as well as extrinsic conflicts including government guidelines and media reporting. Further detail on insights of mixing methods are given in the Mixed Methods Results section as well as the Discussion.

**References**

1. Gugsa F, Karmarkar E, Cheyne A, Yamey G. Newspaper coverage of maternal health in Bangladesh, Rwanda and South Africa: a quantitative and qualitative content analysis. BMJ open. 2016 Jan doi:10.1136/bmjopen-2015-008837
2. O’cathain A, Murphy E, Nicholl J. The Quality of Mixed Methods Studies in Health Services Research. *Journal of Health Services Research & Policy*. [Online] SAGE Publications; 2008;13(2): 92–98. doi:10.1258/jhsrp.2007.007074
