# Supplementary material for: Caregiver perceptions and experiences of paediatric emergency department attendance during the COVID-19 pandemic: A mixed-methods study
Source: PLoS One. 2022 Nov 16;17(11):e0276055. doi: 10.1371/journal.pone.0276055 (PMC9668109; doi:10.1371/journal.pone.0276055)
Supplement: Attachment — Submitted filename: Response to Reviewers.docx [file pone.0276055.s005.docx]

**Manuscript submitted for review to PLOS ONE (PONE-D-21-33341)**

**Author’s response to reviewer’s comments**

We thank Reviewers 1, 2 and 3 for their supportive and constructive comments to help strengthen this article. We detail our comments below and refer to the page numbers of the manuscript file with tracked changes to view these edits.

The major changes in this revised manuscript are as follows:

1. **Introduction**
   a. Addition of literature on paediatric emergency department utilisation during the pandemic in other geographical settings
   b. Further detail on the Watson *et al.* study which explored delays in care-seeking during the pandemic in the UK
2. **Materials and Methods**
   a. Refinement of information presented on the study setting (Lewisham and Greenwich)
   b. Further detail on sampling approach to both studies and responses to invite to participate
   c. Further detail on the quantitative analysis (including use of ANOVA test) and design of the qualitative study (and topic guide) as well as historical data used
3. **Results**
   a. Reformatting of Tables 1 and 2 (with original Table 2 now provided in S4)
   b. Detail from the ANOVA test added
   c. Removal of Fig 1 (as this is to be included as an attachment to the submission to meet journal requirements)
   d. Refinement of qualitative results based on feedback from peer reviewers
   e. The addition of a “mixed-methods results” section to fully integrate our triangulated data to meet the GRAMMS checklist requirements
4. **Discussion**
   a. Addition of reflections on participant behaviour changes based on the timing of interviews during the pandemic
   b. Further description of future research opportunities as well as recommendations for policy and planning, with comparisons made to the existing literature
   c. Additional reflections on the limitations of the study design and methods used
   d. Addition of sub-heading for ‘future research and study implications’
5. **General**
   a. Revisions made to Supplementary Files 3 and 4 – specifically formatting of the document to include manuscript details as well as additions to S3 on the mixed-methods approach to the study and data analysis. Also, the removal of Table 1 in S4 (now in the main manuscript file) and reformatting of Tables in response to reviewer comments. We have submitted clean versions of the files (without tracked changes)

**Reviewer comments and author responses and revisions**

| **Reviewer 1 comment** | **Author’s response and revisions (line numbers according to clean copy)** |
| --- | --- |
| Introduction When identifying gaps in the literature, the authors state “as the pandemic continues there is a need to understand whether the same patterns of behaviour are being seen in emergency care, particularly whether young children are experiencing unintended consequences due to delayed presentation”. However, it is unclear how the present design and results identifies unintended consequences so I would suggest re-wording this as it may be misleading for the reader. | This has been revised to acknowledge that our study is not designed to identify unintended consequences and has been removed for clarity (see lines 107-114).    It is designed to show or demonstrate the gap in the literature around if the issues previously identified are still in existence. |
| Methods Study Design and study population: The authors state that the mixed methods approach was selected because it “allowed for corroboration, triangulation, exploration of complementary and diverging points as well as expansion of ideas”. However, it is not clear from the results how this was carried out as it appears that the quantitative and qualitative data were analysed separately. It could be that I’m interpreting this incorrectly but in that case, this section needs to be re-worded. | An additional segment has been added to the results specifically integrating the quantitative and qualitative results in line with a parallel convergent mixed method approach where integration of the data occurs in the analysis stage (see lines 546-616). |
| Were all attendees to the ED during the timeframe described asked to participate?  If not, how were they selected and was there a risk of any bias?  What was the response rate? | The recruitment process has been made clearer. It now details how the study recruitment evolved from a planned probability systematic sampling to a non-probability convenience sampling method in the methods section (see lines 142-166).  The limitations of this change in recruitment have been discussed in the discussion section specifically about the risk of introduction of bias (see lines 697-703). |
| More information of the design of the survey is needed. Was it piloted ahead of the study?  Any information on validity? | The design of the survey looking at questions asked was explored later in the methods, but this wording has now been moved to the design section.  The survey was designed by the research team and not piloted beforehand; this is discussed in the limitations section in more detail alongside how face validity was not achieved because of this omission. The interview guide was reviewed by an expert team and piloted beforehand. All of this is detailed in the Methods (see lines 169-186). |
| The authors mention that triage scores and outcomes were noted. Were these extracted from medical notes? If so, this needs to be stated as a source of data. | This has been removed from the study as a separate data source as it was felt to not be adding to the overall story. |
| Were any sample size calculations carried out? | For the quantitative component, 150-200 survey respondents were expected based on attendance figures in March/April.  For the qualitative components, we aimed to continue interviewing until theoretical saturation was met.  This is detailed in the Methods (see lines 163-166). |
| I think Table 1 in S4 should be a table in the main text rather than a supporting file. | This has been moved and formatted to meet journal requirements. |
| Historical figures: What was the source of the data described in this section? Are these figures publically available or were they extracted from a hospital database for the study? They form the basis of a key conclusion in the study so I think it’s important to include a section in Methods to outline the data source. | The historical data sources were accessed from the Business Team and have been identified in the manuscript (see lines 188-190). In addition, these sources are not available to the public and this has been stated also. (see lines 188-190). |
| Page 12: Please use the ρ coefficient to denote the results of Spearman’s rho instead of “calculated correlation co-efficient” | This has now been specified in the paper (see line 254). |
| Page 13, line 629: “appear outweighed” This phrasing is difficult to understand. | This sentence has been changed to make it clearer that even if caregivers have concerns these are overridden and replaced by the dual influences of how their child’s health and advice/ reassurance from others can overcome these worries and support their attendance decision. |
| One element that I feel is missing from the research is information on the socioeconomic profile of the parents (education, job status, income etc.) as this is well known to influence health seeking. I think this is an important limitation and needs to be recognised or fleshed out in the discussion. Maybe information about the location of or population the hospital serves? | In Methods section information about socioeconomic factors have been added in (see lines 137-139).  In discussion section a line has been added around the socioeconomic profile for future studies with further evidence provided around how different subgroups have been affected in the pandemic (see lines 729-734). |
| This research took place in November 2020 when the pandemic had been ongoing for 9 months and people had had time to adjust to living in a pandemic, restrictions, mask wearing etc. Is it possible that this may have had an impact on the results? | We have discussed the temporal nature of the findings in the Results section, however, have added additional sentences in the Discussion section (under key findings) (see lines 628-632). |
| Are there any recommendations for policy or planning arising from the research? This would be particularly valuable with regards to future planning for public health emergencies. | The following recommendations have been added in based on our study and also from reviewing the literature around public health messaging in emergency situations:   - Tailoring specific information to groups / audiences - Engagement of community leaders - Rectifying any miscommunications early - Medical professionals being a trusted source |
| Table 1: It’s not clear why you would expect there to be a difference between those who completed the questionnaire only, interview only or both. I think this should be collapsed into one column and this table brought into the main text. | The purpose of the table was to delineate that there was no difference. However, on reflection we have realised this may be confusing for readers and given we have the separate table of caregivers who participated in the interview the reader should be able to infer there is no difference between the two groups and so we have acted on your suggestion. |
| Table 2: As mentioned previously, the source of this historical data needs to be included in the methods | The source of the historical data has been identified as contacting the Business Analysis Team and we have outlined this is not available to the public. |
| Table 3: As with table 1, it’s not clear why you would expect there to be a difference between the two groups. As a result, I don’t think Table 3 is needed at all and would recommend removing. | As outlined previously the idea of including the results was to show no difference between the groups. We have decided to keep the data to support the findings reported in the main manuscript but agree it should remain in the Supplementary File. |

| **Reviewer 2 comment** | **Author’s response and revisions (line numbers according to clean copy)** |
| --- | --- |
| 1. To start, a general comment of editing. This paper needs to be proofread, as there are several grammatical errors in the paper. Please attend to this. | We have proofread the paper. |
| 2. The abstract reads well, but the conclusion is vague (also in the paper). Please clarify what the take-home message is of the paper and what we can learn from the your findings. What should policy-makers, health workers or parents do differently? | We have revised the Conclusion section of the Abstract on page 3 and the Conclusion section in the main text on page 39 to better communicate our recommendations. However, the purpose of this manuscript is to report findings and it is beyond the scope of this study to dictate specific messages tailored to different groups to improve care seeking. The suggestions we do make are grounded in the data- for instance, that caregivers should not delay care seeking, emphasising the safety of hospital spaces in terms of SARS-COV-2 transmission. |
| 3. The introduction is well-written, but some of the studies mentioned in the background do not show a significant delay in seeking care for children. This does not reinforce your objectives of doing this study. Is there anything else in the examples you can highlight that are of interest to your study? This links to my concerns about the overall objectives of the study, as the background does not stress the concerns at length, and therefore, you need to reinforce your argument with using other examples. For instance, what were the qualitative outcomes in reference 26? And how are these different or similar to your work? | We have added references indicating significant declines in global settings including Germany [8], Ireland [9] and Greece [10]. We have included the studies that do not show significant delays to emphasise that the picture remains mixed and context-specific which highlights why our study is needed for this specific population.  We agree that including some more information on the qualitative outcomes from the Watson *et al.* 2021 paper [now reference 29] would be helpful, so we have this in the Introduction. We have reflected on our findings compared with Watson’s paper the Discussion. |
| 4. The methods are well-described, but I was wondering why data collection was only conducted during office hours and during the week? Maybe parents coming in the weekend or evening might have delayed attending the emergency room for different reasons or waited longer? | We have referenced that this was a limitation to our study in the limitations section.  Historically research has only been conducted in hours 9-5 at Lewisham and Greenwich NHS trust in the paediatric department due to the contractual obligations of the research team. Whilst this research was conducted voluntarily, by those involved in data collection, for uniformity and in line with previous studies the recruitment occurred during ‘office-hours’. |
| I am also curious to read more about the questions asked in the interview, you only mention information provision as a topic, which seems limited.  I would also like to read which emerging themes derived during the data analysis process.  I would also add the specifics of the participants and the length of the interviews to the methods, instead of the findings.  Also, the interviews were really short, was this time sufficient for the researchers? And why, or why not? | The interview topic guide which lists the questions asked during the interviews is provided in the Supplementary Information (Item 2).  The themes which emerged from the data analysis are those which are presented as headings and described in the Results section of the manuscript, also, an overview of these are depicted in Figure 1.  Thank you for the suggestion of moving this table to the methods. After some deliberation we decided to keep the participant characteristics and nature of interviews (including length) in the Results section to be consistent with the presentation with the quantitative findings. We feel that this best supports the narrative and flow of the paper.  The interviews ranged from 10-45 minutes. As the interviews were conducted in a busy Paediatric A&E department whilst participants’ children were waiting or in between receiving emergency care, the length of time that participants were willing to participate in an interview varied and may have been a shorter length than if they were interviewed at a less urgent time. Despite this, as the richness and range of the results illustrate, valuable findings emerged from the interview data. |
| 5. The findings are relevant, but I wonder if you can shorten theme 1. This speaks about the general experiences of COVID-19 and has some interesting quotes, but they are not related to the main topic. I would emphasize the findings of Theme 2 more, as they link well to your research questions and are well described. The theme also gives a gives a good impression of the confusion and fear of parents and their decision-making process of seeing a health care provider for their children and trying to keep other family members safe. We can learn a lot about that.  In Theme 3, I was expecting to read more about parents’ interactions with health workers, such as being judged for coming in or feeling inadequate as parents, but they are not described in the theme. Only the waiting times were discussed in the theme, which seems limited. | We agree with the comment made by Reviewer 2 and have shortened theme 1.  We agree and have emphasised theme 2 more in the Discussion.  Exploring the interactions of caregivers and healthcare workers was not within the scope of the study (nor interview topic guide) and did not emerge as a theme from the interviews. |
| 6. The discussion of the findings is limited, which might be attributed to the novelty of the study, but I would have liked to see a comparison of your findings with studies conducted in other countries.  There is literature around delayed decision-making for getting immunisations for children for instance, which has been studied in several countries.  I also have several remaining questions that seem relevant, including; how were other paediatric departments in government clinics attended in other countries, or parts of the UK?  And what can we learn from your findings?  If we get into a similar situation, like COVID-19, how can paediatric communication be improved? Or what should be teach young parents about attending care during a national crisis. How can you convince parents that they are not ‘overburdening the NHS’ and reassure them that their own health and that of their children is most important? Overall, I think this study is relevant and results should be published, but several improvements need to me made to strengthen the aim, objectives and outcomes of the paper. | We have added findings from other global studies in the Introduction section [8-10] and have added an additional reference [43] in the Discussion to unpick how caregiver characteristics may explain delayed presentations to PED.  We have added detail on the description of PED attendance in Greece in lines 69-73 and existing literature in the UK/England in lines 80-90.  We have added recommendations for risk communication in lines 729-757 as well as conclusion section. |
| **Reviewer 3 comments** | **Author’s response and revisions** |
| Methods: More information should be provided here,  eg: method of selection of participants (sampling technique), method of data analysis/ analysis carried out | We have addressed and added more clarity around the sampling technique used in the study. |
| Result: Quantitative result appears too scanty here, add a little more details eg demographic characteristics. | We have restructured the results section and added a subheading and results to refer to demographics.  We have re-worked Table 2. We have also added additional analyses using ANOVA test to compare mean ages between caregiver ethnicity |
| Methods: Line 127, Kindly provide the name of the study design used in this study, perhaps read up types of study design and identify where this falls under. What was written is more of a description of what was done. | Thank you for prompting us to be more explicit with the type of study design used. It is a mixed methods parallel convergent study design. This is stated in lines 117-122. |
| Results of quantitative survey should also be presented in tables. | Please see Supplementary File 4. |
