# Supplementary material for: Caregiver perceptions and experiences of paediatric emergency department attendance during the COVID-19 pandemic: A mixed-methods study
Source: PLoS One. 2022 Nov 16;17(11):e0276055. doi: 10.1371/journal.pone.0276055 (PMC9668109; doi:10.1371/journal.pone.0276055)
Supplement: Attachment — Submitted filename: Response to Reviewers.docx [file pone.0276055.s006.docx]

**Caregiver perceptions and experiences of paediatric emergency department attendance during the COVID-19 pandemic: a mixed-methods study**

Dr Gayle Appleby^¶^, Vasiliki Papageorgiou^¶^, Dr Shona Horter, Alexandra Wharton-Smith, Dr Tina Sajjanhar, Dr Anthony Hemeson, Dr Emmanuel Singogo, Dr Benjamin Cahill, Dr Sophie Keers, Dr Lorraine Wicksey, Dr Marylyn Emedo, Dr Alastair Yim, Dr Maggie Nyirenda-Nyang’wa*

^¶^ indicates co-first authorship

* corresponding author

**Manuscript submitted for review to PLOS ONE (PONE-D-21-33341R1)**

**Author’s response to reviewer’s comments**

We thank Reviewer 3 for their additional comments. We outline our responses below and refer to the line numbers of the manuscript file (with tracked changes) to view these edits.

The major changes in this revised manuscript are as follows:

1. **Introduction**
   a. Minor edits clarifying the findings from the Watson *et al.* study which explored delays in care-seeking during the pandemic in the UK
2. **Materials and Methods**
   a. Further detail on how the quantitative and qualitative data were analysed and integrated - including new references 44-46 and a new Figure (Figure 2) demonstrating how the data are jointly integrated
   b. The addition of a sub-section on positionality and reflexivity including the addition of a new reference (48).
3. **Results**
   a. The addition of 5 new quotes under various sub-themes in Theme 1
   b. The addition of 2 new quotes under various sub-themes in Theme 2
   c. Further refinement of Mixed-Methods results section outlining how the quantitative and qualitative data compare and contrast (including complementarity) including new references 44-46
   d. Addition of six quotes to illustrate various concepts presented in both the quantitative and qualitative results (under Mixed-Methods heading)
4. **Discussion**
   a. Minor amendment made to wording under the future research and study implications subsection
5. **General**
   a. Revisions made to Supplementary File 4 – specifically the removal of the qualitative table of demographic identifiers for the interview participants due to concerns about potential identification. A further the addition of Table 3 which displays the mixed-methods integration of data on ‘level of worry’. We have submitted clean versions of the files (without tracked changes)
6. **Journal Requirements**
   a. Previously we had identified the Cambridge and Herts ethic team would act as a point of contact for any data set requests. This will now be the Lewisham and Greenwich Research and Development Team (LGT RND) [LH.RD@NHS.NET](mailto:LH.RD@NHS.NET) subject to ethical approval.

We include our responses to the reviewers' comments below.

We wish to resubmit this research article titled “**Caregiver perceptions and experiences of paediatric emergency department attendance during the COVID-19 pandemic: a mixed-methods study”** and thank you for your consideration of this manuscript.

Yours sincerely,

Gayle Appleby

**Journal requirements and author responses and revisions**

| **Academic editor comment** | **Author’s response and revisions** |
| --- | --- |
| Please review your reference list to ensure that it is complete and correct. If you have cited papers that have been retracted, please include the rationale for doing so in the manuscript text, or remove these references and replace them with relevant current references. Any changes to the reference list should be mentioned in the rebuttal letter that accompanies your revised manuscript. If you need to cite a retracted article, indicate the article’s retracted status in the References list and also include a citation and full reference for the retraction notice | This has been done all references are correct and only one reference modified, several have been added in for the explanation of the mixed methods analysis. No articles we cite have been retracted. |
| While revising your submission, please upload your figure files to the Preflight Analysis and Conversion Engine (PACE) digital diagnostic tool, [https://pacev2.apexcovantage.com/](https://eur01.safelinks.protection.outlook.com/?url=https%3A%2F%2Fpacev2.apexcovantage.com%2F&data=05%7C01%7Cmaggie.nyang%27wa%40ucl.ac.uk%7C9e93b094e5e54f30d26308da6bf20637%7C1faf88fea9984c5b93c9210a11d9a5c2%7C0%7C0%7C637940984477461831%7CUnknown%7CTWFpbGZsb3d8eyJWIjoiMC4wLjAwMDAiLCJQIjoiV2luMzIiLCJBTiI6Ik1haWwiLCJXVCI6Mn0%3D%7C3000%7C%7C%7C&sdata=IZK8r1CsP6JDohX9NLj22xOgQDp%2Fmcfp35t0UvzFq8s%3D&reserved=0). PACE helps ensure that figures meet PLOS requirements. To use PACE, you must first register as a user. Registration is free. Then, login and navigate to the UPLOAD tab, where you will find detailed instructions on how to use the tool. If you encounter any issues or have any questions when using PACE, please email PLOS at [figures@plos.org](mailto:figures@plos.org). Please note that Supporting Information files do not need this step. | No changes made to Figure 1.  We have uploaded Figure 2 to PACE and checked it meets PLOS requirements. |

**Reviewer comments and author responses and revisions**

| **Reviewer 3 comment** | **Author’s response and revisions (line numbers according to revised copy)** |
| --- | --- |
| Overall comment: I think this paper has drastically improved and I am happy with the changes made in the updated version. I am happy for this paper to be accepted, although I have made some additional suggestions below. Once these are considered, I think this paper will make an excellent contribution to body of knowledge focusing on the impact of the COVID-19 pandemic in the global North. |  |
| 1. Abstract: in the methods, you speak about data triangulation, but in the mixed methods study, you only used 2 different methods; survey and interviews. How was the data triangulated?   And more importantly, how was the data analysed and synthesized? | On reflection we have removed the word triangulation from the methods section and replaced this with comparison (line 29). It was meant to represent the concept of corroboration between two sets of data; however, upon further reading around triangulation in the context of MMR research we realise that this is confusing and doesn’t reflect the term as per Denzin and Lincoln 1998.  Thank you for asking us to explain how the data was analysed and synthesized. We hope that the explanation is clearer now.  We include additional detail on how the data were converged in the Methods section (lines 224-231) with the inclusion of 3 new references to support these statements (refs 44-46).  We have also included an additional Figure (2) exploring how the results were combined and further detail on these comparisons in the Mixed-Methods Results section in lines 631-650. |
| 2. Line 67: use a different word to start the sentence. Despite this – does not link well to the previous paragraph, unless you say: ‘despite hospitals remaining open throughout the various lockdowns, the was a decreased …..’ | Text modified (lines 67-68) as per Reviewer 3 suggestion to improve the link to the previous paragraph  ‘Despite hospitals remaining open during this first lockdown, there was a decreased number of children attended paediatric emergency departments in the UK during this period (5).’ |
| 3. Line 102: ‘was driven by fear and knowledge’ – can you be more specific here? What kind of knowledge are we speaking about…. Or a lack of knowledge maybe? This seems relevant to the study. | We have modified the text (lines 100-105) to reflect the weight given to the study’s conclusions by the authors. As identified it was a lack of knowledge that was one aspect, but fear was the main driving force.  This study by Watson *et al.* (29) identified that the delay in decision to care-seeking was in part driven by a lack of knowledge but primarily fear. Several factors influenced this fear, including acquiring COVID-19 or overburdening hospitals. Identified factors that could mitigate against the fear and lack of knowledge included: adequate signposting, access to decision-making aids and drawing support from others. |
| 4. Line 161: consider rewriting: ‘that they were too worried about and wanted to focus on their child.’ Just change into; feeling too worried about their child. | Thank you for the suggestion we have modified the line (line 161) as suggested.  Additionally, we have included further detail on worries about children influencing hospital attendance in the Results where qualitative and quantitative data are combined and compared (lines 684-693). |
| 5. Line 188: I understand that the data cannot be disclosed, but how was the ‘historical data’ used? To see if the child had been attending the ER before COVID, or to understand illness and symptoms? | We have tried to make this section clearer (lines 188-192) that this was a total review of attendance figures and not relating to whether the child had attended ED before COVID.  The hospital business analysis team was contacted relating to attendance figures (available in the public domain) and other parameters for a limited time period 2019 and 2020 **(S4)**. For access to internal, historical data (not available in the public domain) the hospital business analysis team was contacted by the research team - relating to total attendance figures, reasons for attendance and other parameters over set time periods in 2019 and 2020 **(S4)**. This allowed for comparison between our study data and previous time periods to review trends **(S4)**. |
| 6. Line 205: what themes derived out of the analysis?   And how was reflexivity and positionality of the project staff being explored and negotiated within the team? I would like to read more about this, as it is an important aspect of data analysis. | We have edited this section to list the 3 themes that emerged from the analysis (lines 206-212).  We agree that a section on positionality and reflexivity would be helpful so have included a new sub-section in lines 231-249. |
| 7. Line 298: I think it would be great to have a quote that illustrates the ‘new normal’. | We agree this would be helpful and have added two new quotes (from participants ID4 and ID10) to display the new normal in terms of how it has affected day to day life and life in the hospital (lines 340-351) |
| 8. Line 309: Similar to comment above. A quote from ID12 or ID20 speaking about the differences in illness symptoms between flu and COVID could strengthen this paper | Thank you for your suggestion around adding in the quote about flu and COVID.  We have referenced both ID12 and ID20 as they provide contrasting views to how people view COVID-19 as just another flu contrasting with something more worrying (see lines 365-370). |
| 9. Line 350: A quote about the confusion of the rules would be a benefit to this paper | A quote from participant ID5 around the confusion of the rules and frustrations of the government has been added (lines 412-417).  There is also a quote later in line 423-426 from ID5 that addresses this. |
| 10. Line 376: A last sentence can be added here that explains that the interviewees had been living with the pandemic for 6 months at the time of the interview, so had a chance to adapt to the ‘new normal’. | A line has been added in thank you for your suggestion (see lines 442-445). |
| 11. Line 395: I would like to read a few quotes here too, especially about the ‘parents’ instinct or ‘gut feeling’. | Two quotes (ID12 and ID18) have been added around parents’ instinct/ gut feeling to illustrate this point in lines 458-463. |
| 12. Line 739 – 741: this sentence does not flow well, and I am not sure what the authors are trying to say. Please rewrite/simplify | We have rewritten the sentence for clarity (lines 863-868). |
